# Evaluation of the Quality of Results of Lung Cancer Surgery in France Using the PMSI National Database

**DOI:** 10.3390/cancers17040617

**Published:** 2025-02-11

**Authors:** Alain Bernard, Jonathan Cottenet, Catherine Quantin

**Affiliations:** 1 Department of Thoracic and Cardiovascular Surgery, University Hospital, 21000 Dijon, France; 2 Department of Biostatistics and Bioinformatics, University Hospital, 21000 Dijon, France; jonathan.cottenet@chu-dijon.fr; 3 INSERM, CIC 1432, Biostatistics, Biomathematics, Pharmacoepidemiology and Infectious Diseases (B2PHI), 2100 Dijon, France; 4 CESP, Inserm, High-Dimensional Biostatistics for Drug Safety and Genomics Paris-Saclay University, 94807 Villejuif, France

**Keywords:** lung cancer, surgery, postoperative complications, quality assessment

## Abstract

**Background**. Given the complexity of lung cancer surgery, this study aims to provide an overview of hospitals authorised to perform lung cancer surgery in France, and to assess their performance focusing on severe post-operative complications and 30-day in-hospital mortality based on the Clavien–Dindo classification (grade > 2). **Methods**. We included all patients (n = 64,304) who underwent pulmonary resection for lung cancer from the French hospital database (2019–2023). To quantify variations within regions, we used the ratio of the 90th to the 10th decile of the standardised outcome rate of the hospitals. We used a hierarchical logistic regression model to estimate the adjusted odds ratio (aOR) according to the number of annual procedures. We then used the results of this modelling to see how the standardised rate estimate might evolve after simulating a new organisation of hospitals authorised to perform this surgery. **Results**. A total of 18,151 patients (28%) had severe complications (Clavien–Dindo > 2). Compared to hospital performing less than 100 procedures/year, the risk of severe complications was significantly reduced for hospitals performing between 101 and 250 procedures/year (aOR = 0.83 [0.77–0.89]) and more than 250 procedures/year (aOR = 0.85 [0.77–0.93]). A simulation of hospital reorganisation, using 100 procedures/year as the threshold value, showed that 477 severe complications could have been prevented over the period. **Conclusions**. This study shows inequalities in performance indicators between hospitals in each French region. The influence of the volume of activity raises questions about the need to restructure the offer of care for complex surgeries, such as lung cancer surgery.

## Simple Summary

Given the complexity of lung cancer surgery, it has become imperative to carry out an in-depth assessment of the current state of these surgical practices throughout France in order to improve the quality of care. This study aims to provide an overview of hospitals authorised to perform lung cancer surgery and to assess their performance based on key outcomes. This innovative work shows the variability of lung cancer surgery outcomes within French regions with complication rates (Clavien–Dindo > 2) up to three times higher between hospitals. A simulation of hospital reorganisation (threshold value = 100 procedures/year) made it possible to estimate that 477 severe complications or deaths could have been avoided over the study period (2019–2023). The clear link between surgical volume and patient outcomes calls for a serious re-evaluation of the current healthcare organisation for complex surgeries like lung cancer resection.

## 1. Introduction

Lung cancer (LC) remains one of the most prevalent and deadly forms of cancer worldwide. While surgery is a key treatment option, only about 20% of lung cancer patients in France are eligible for surgery due to the advanced stage of the disease at diagnosis or poor general patient health. This reflects the challenges associated with disease progression and the patient’s overall condition, limiting the potential for surgical intervention [1]. LC surgery requires specialised care, ideally provided by dedicated hospitals with expertise in thoracic oncology. The French government launched the first ‘Cancer Plan’ in 2009 [2], which radically reformed cancer care, one of the key measures being the requirement for hospitals performing cancer-related surgery to obtain specific authorisation. Under the plan, hospitals were required to perform a minimum of 30 LC related procedures/year in order to obtain authorisation. Recently, this threshold was raised, with hospitals now having to perform at least 40 procedures/year to retain their authorisation [3]. At present, 146 hospitals across France are authorised to perform thoracic cancer surgery [3]. The quality of the results obtained by the teams performing these surgeries has been relatively little studied in France, unlike in other European countries and the United States [4,5]. Given the complexity of LC surgery, it has become imperative to carry out an in-depth assessment of the current state of these surgical practices throughout France in order to improve the quality of care, and in particular to reduce the frequency of severe post-operative complications. This study aims to provide an overview of the hospitals authorised to perform LC surgery, and to assess their performance on the basis of key outcomes. The specific objectives of this work are as follows. Firstly, we aimed to describe the characteristics of all hospitals in France that perform LC surgery, including public or private status, their distribution across the 13 French regions, and the characteristics of the patients they admit. Our second aim was to assess the surgical performance of these hospitals, using the Clavien–Dindo grade > 2 classification (which includes severe post-operative complications and 30-day in-hospital mortality) as the main outcome indicator.

## 2. Materials and Methods

### 2.1. Database and Inclusion

This study utilised the French national hospital database from the Programme de Médicalisation des Systèmes d’Information (PMSI), which includes discharge abstracts for all inpatient admissions to both public and private hospitals across France. Diagnoses recorded during the hospital stay are classified according to the 10th edition of the International Classification of Diseases (ICD-10) [6,7], while medical and surgical procedures performed during hospitalisation are coded according to the French Common Classification of Medical Procedures (CCAM). We included patients from this database who underwent pulmonary resection for LC in France between 2019 and 2023. Specifically, we selected patients with a primary diagnosis of LC (ICD-10 codes C34), along with a corresponding surgical procedure for LC (CCAM codes) performed during the same hospital stay [8,9].

### 2.2. Patient Characteristics

For each patient, we collected data on age, gender, and surgery-related factors, including the type of surgical approach (thoracotomy, video assisted thoracic surgery (VATS) or robot-assisted surgery) and the nature of the resection (limited resection, lobectomy, bilobectomy or pneumonectomy). Additionally, we considered comorbidities, such as various diseases (pulmonary, cardiovascular, peripheral vascular, liver, neurological, kidney, hematologic, metabolic, infectious), cerebrovascular events, anaemia and medications (preoperative chemotherapy and steroids). We also calculated a modified Charlson Comorbidity Index (CCI) to assess the overall comorbidity burden [10].

### 2.3. Region and Hospital Characteristics

In France, hospitals are classified into several categories, including academic (teaching), non-academic, non-profit private, or private. For each hospital, we determined the number of pulmonary resections performed per year.

The hospitals were located in one of the 13 regions of metropolitan France: Auvergne-Rhones-Alpes (ARA), Bourgogne-Franche-Comté (BFC), Bretagne (BRE), Centre Val-de-Loire (CVL), Corse (COR), Grand-Est (GE), Hauts-de-France (HdF), Ile-de-France (IdF), Normandie (NOR), Nouvelle Aquitaine (NA), Occitanie (OC), Pays de la Loire (PdL), and Provence-Alpes-Côte d’Azur (PACA).

### 2.4. Outcome (Quality Indicator)

Our main outcome was based on the Clavien–Dindo classification [11], which was transformed into a binary variable. The variable was equal to 1 if the Clavien–Dindo classification was higher than 2. This grade > 2 rating included severe post-operative complications and 30-day in-hospital mortality (Table S1).

Severe post-operative complications were defined as the presence of one or more of the following postoperative conditions [11,12]: pain, parietal complications (wall abscesses or wall haematomas), tracheostomy, reintubation, adult respiratory distress syndrome, bronchopleural fistula, empyema respiratory failure, arrhythmia, malnutrition, phlebitis, pleural effusion, pulmonary embolism, pneumonia, bleeding requiring re-operation, myocardial infarction, stroke, ischemia of the lower limbs, septicaemia, and heart failure.

Thirty-day in-hospital mortality was defined as all deaths occurring during the same hospital stay as the operation or within 30 days of the operation.

Hereafter, we will refer to this outcome (Clavien–Dindo > 2) as “severe complication”.

### 2.5. Statistical Analyses

The number of annual procedures for each establishment was categorised into 3 quantiles: <100, 101 to 250 and >250 procedures/year.

To estimate the standardised outcome rate (Clavien–Dindo > 2), we used an indirect method by including the following variables in the logistic regression model: age, gender, comorbidities, CCI, type of pulmonary resection, surgical approach and year. To describe regional variations in the number of procedures and the standardised outcome rate, we used the median, the 1st quartile and the 3rd quartile. To quantify variations within regions, we used the ratio of the 90th to the 10th decile of the standardised outcome rate of the hospitals. We used a hierarchical logistic regression model to estimate the adjusted odds ratio (aOR) for each of the three classes of number of annual procedures and type of establishment, with their 95% confidence interval. We also included patient characteristics, type of procedure and year in the model. These models are used to estimate unexplained heterogeneity between regions, indicated by the inter-regional variance [13,14]. These models were also performed for limited resections and lobectomies as sub-analyses.

Finally, we used the results of this modelling to see how the standardised rate estimate might evolve after simulating a reduction in the number of hospitals authorised to perform this surgery. We used the estimated standardised rate using the logistic regression model explained above to obtain the number of events expected in each region if only centres performing more than 100 procedures/year were selected. Finally, the number of avoidable severe complications or deaths was calculated as the difference between the observed number of complications and the expected number of events.

The calculations were carried out using STATA V.18 statistical software (StataCorp, College Station, TX, USA).

### 2.6. Ethics, Patient and Public Involvement

Patients and the public were not involved in this study. As this was a national retrospective analysis using pseudonymised data (no personally identifiable information), patient consent was not applicable. The French national hospital database does not contain any identifying details: patient identities are pseudonymised, allowing data linkage for each individual without revealing their identity. Ethics approval for the use of this database was granted by the French National Commission for Data Protection (declaration of compliance with reference methodology 05 obtained on 7 August 2018 under number 2204633 v0), and this study adhered to the tenets of the declaration of Helsinki.

## 3. Results

From 2019 to 2023, 64,304 patients underwent lung resection for cancer. The number of surgeries increased over time (2019: 12,367 patients, 2023: 14,227 patients, Table 1). A total of 18,151 patients (28%) had a severe complication (Clavien–Dindo > 2), which was used as a quality indicator. Over the study period, we observed a decrease in severe post-operative complications, as well as in mortality (Clavien–Dindo 5), which fell from 2.3% in 2019 to 1.5% in 2023 (Table 1).

### 3.1. Regions and Characteristics of Hospitals

A total of 171 hospitals were performing lung resections for cancer during the study period. The median number of procedures/year in France was 53, with interquartile ranges of 16 and 101. By region and depending on the size of the region, the number of hospitals ranged from 2 in Corsica to 24 in Rhône-Alpes-Auvergne (Table 2). However, the number of hospitals was not proportional to the size of the population in the region. For example, 24 hospitals performed lung resections in Rhône-Alpes-Auvergne, compared to only 19 hospitals in the more populous Ile-de-France region (Table 2). The number of annual procedures performed by hospitals in the different metropolitan regions is shown in Table 2. The medians ranged from 20 in Corsica to 121 in Grand-Est. In most regions, 25% of hospitals performed fewer than 20 procedures/year.

### 3.2. Standardised Rate of Severe Complications (Clavien–Dindo > 2) in the Current Situation: Estimation and Modelling

**Figure 1:**
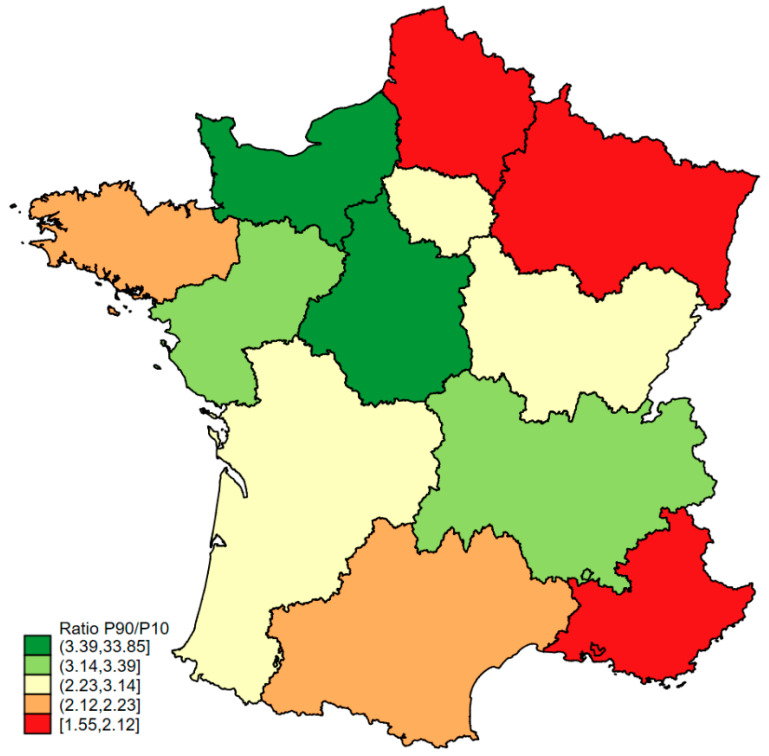
Standardised rate of severe complications (Clavien–Dindo > 2) in metropolitan regions.

Supplementary Figure S1 shows the distribution of the median standardised complication rates for hospitals in the different regions. Regions exceeding the national rate of 28% included Centre-Val-de-Loire, Corsica, Grand-Est and Ile-de-France. The 90/10 inter-decile ratios for the regions are shown in Figure 1.

The inter-decile ratio was greater than 3 in Normandy, Centre-Val-de-Loire, Pays de la Loire and Rhône-Alpes-Auvergne, meaning that the rates of severe complication were up to three times higher between hospitals. For the other regions, ratios ranging from 2 to 1.55 suggested that the inequalities were less marked. The classification of hospitals into 4 quantiles of standardised complication rates is shown in Table 3. In the regions of Centre Val-de-Loire, Corsica, Grand-Est, Ile-de-France, Nouvelle Aquitaine and Provence-Alpes-Cote d’Azur, most hospitals fell into the classes with a high rate of severe complications. In the other regions, the distribution was fairly balanced between the four classes, except for Pays de la Loire, where most hospitals were in the first class with a low rate of severe complications.

The number of annual procedures, divided into three classes, was included in the hierarchical logistic regression model used to estimate inter-regional variance (Table 4). After adjusting for comorbidities, surgical approach, and type of resection, we found that teams from hospitals performing more than 100 lung resections per year reduced the risk of having a severe complication or post-operative death by 20% compared with hospitals performing fewer than 100 procedures/year (aOR = 0.83 [0.77–0.89] for hospitals performing between 101 and 250 lung resections per year and aOR = 0.85 [0.77–0.93] for hospitals performing more than 250 procedures/year, Table 4 and Table S2). Private not-for-profit and private for-profit hospitals had respective aORs of 1.35 [1.19–1.52] and 1.10 [1.01–1.19] compared with non-academic hospitals (Table 4). The median OR was 1.132, showing moderate heterogeneity between regions, taking into account the same patient and hospital characteristics. Sub analyses for lobectomies and limited resections showed similar results: the threshold of 100 procedures was significantly associated with a reduction in postoperative outcomes for lobectomies (*p* < 0.01, Table S3) and we observed a trend towards this reduction for limited resections (*p* = 0.06, Table S4).

### 3.3. Estimate of the Standardised Rate of Severe Complications (Clavien–Dindo > 2) After Simulation of a Reorganisation of Hospitals

Following the results of our model, we simulated a reorganisation of the care offer, using 100 procedures/year as a threshold value for hospitals to be authorised to perform LC surgery. If this were the case, only 44 hospitals would have been performing this type of surgery in France. In this hypothetical situation, 477 severe complications or deaths would have been avoided.

## 4. Discussion

This study, conducted from 2019 to 2023, included all French hospitals (171 hospitals) performing lung cancer surgery. While the differences in severe complication (Clavien–Dindo > 2) rates between regions are minor, the variations between hospitals within those regions are significant considering that complication rates were up to three times higher in certain hospitals. We found that the primary factor explaining this variation was low surgical volume and that the results were much better for hospitals carrying out more than 100 procedures/year. After observing that many hospitals had a low surgical volume (in most regions, 25% of hospitals perform fewer than 20 procedures/year), we simulated a reorganisation of care in which hospitals would reacquire a threshold of 100 procedures/year to be authorised to perform this surgery. In this simulation, which reduced the number of authorised hospitals to 44,477 severe post-operative complications or deaths could have been avoided over the 2019–2023 period.

This study assessed the results of LC surgery, showing considerable variability in quality of care between hospitals. Our results demonstrate that there is a need to change how LC surgery is offered in France. In particular, centralisation would be an appropriate means of ensuring that hospitals have sufficient volumes of activity. This type of study has not been carried out in France so far [15,16,17,18].

According to a literature review previously published by our team, the standardised 30-day mortality rate for LC surgery is higher in France than in most other European countries. In light of our findings, it is worth considering whether these differences can be partly explained by the dispersion of surgical teams in France, some of which have low annual case volumes. For instance, when we compare England and France, which have similar populations, only 27 hospitals in England are authorised to perform lung resections versus 146 in France [19].

Many countries have already recognised the need to consolidate the hospitals performing these surgeries, a process known as regionalisation. For example, Denmark, Sweden, Finland, England, Norway, Portugal, and Ireland have regionalised surgical facilities for complex operations. Other countries, like Austria, Germany, Switzerland, the Netherlands, and Belgium, have opted to increase the minimum threshold required to obtain authorisation. In all these countries, the current minimum threshold is higher than in France [20,21,22]. Even if the authorisation threshold for cancer surgery was recently raised from 30 to 40 procedures/year [3] in France, it remains comparably low.

This study appears to confirm the detrimental effect of the dispersion of LC surgery on quality of care, especially since the distribution of centres is not even. Differences in hospital outcomes within regions may have two main causes: patient frailty and quality of care. However, when estimating the standardised rate, we took into account patient comorbidities, age, gender and CCI. We therefore believe that the observed heterogeneity is mainly due to the quality of care.

The principle of consolidating hospitals to provide high-performing surgical facilities that benefit patients is supported by numerous publications showing that the volume of surgical activity correlates with the quality of care. For example, in a previous study, we identified a significant relationship between hospital volume and 30-day mortality [23]. Furthermore, it has been shown that the dispersion of hospitals creates inequalities in patient access to new technologies. For instance, teams that perform few surgical procedures struggle to implement robotic surgery programs. These programs require surgeons to undergo adequate training, which is difficult to achieve effectively if the surgical activity is too low.

We believe that the consolidation of surgical facilities can only be successful if it is carried out on a regional scale, following consultation with relevant stakeholders, and taking into account the specific needs of the population, particularly their socio-demographic characteristics. The designated hospital should include high-performing surgical facilities, both in terms of operating rooms and intensive care units, with access to new technologies. Another crucial criterion is the competence of the medical and surgical teams, including anaesthesiologists, radiologists, intensive care givers, nurses, as well as multidisciplinary collaboration. Consolidation could make selected hospitals more attractive to practitioners, offering them the opportunity to train and work within competent multidisciplinary teams. This obviously raises the question of the links between specialists and general practitioners. Consolidation concerns not just surgeons, but all health professionals involved, and it therefore needs to be analysed from two points of view. The first is the point of view of care providers: beds would have to be ‘moved’ from low-activity hospitals to high-activity hospitals, which would mean relocating all the medical and paramedical teams and their families (changing the spouse’s professional situation, moving schools for the children), enlarging buildings, etc. The second point of view involves patients, and strengthens the role of the family doctor, who must be able to direct patients quickly and easily to dedicated centres. This involves explaining to patients that ‘travelling further’ will enable them to receive ‘better care’.

One of the strengths of our study is the large sample size, which comprises 64,304 patients. Our national administrative database is an invaluable resource for evaluating the quality of care, as it collects detailed patient information from all hospitals in France, with nationwide recruitment over a five-year period. It offers a comprehensive source of epidemiological data on hospitalised patients in France [24,25]. Furthermore, the data related to pulmonary resection for LC are sufficiently reliable, allowing for precise identification of these patients, as demonstrated in previous studies [9].

The limitations of our study are primarily related to the use of ICD-10 codes for patient selection and outcome assessment. Misclassification or underreporting of biases, especially regarding comorbidities, is a potential limitation of this study. Coding practices may differ between hospitals, as diagnoses can be recorded by either clinicians or information system technicians. However, the quality of coding is routinely audited in a standardized manner by medical information professionals at each hospital to ensure the accuracy of diagnoses and enhance the documentation of comorbidities. We also believe that surgeons preferentially code segmentectomies as lobectomies to obtain a better valorisation. We did not analyse complications specifically by type of surgery but performed an overall analysis for all lung resections. Therefore, this issue of coding of resections has no influence on the results presented in the article and cannot constitute a major statistical bias. It is also conceivable that there could be significant differences in the severity of illness between hospitals, which could impact the analyses of mortality and severe postoperative complications. Nevertheless, we used a multilevel analysis model to account for variability between hospitals. In addition, over-reporting of milder forms of complications would not have a significant impact on our results, since we focused on severe complications.

Another limitation concerns the tumour, node, metastasis (TNM) stage, which can influence mortality but cannot be recorded in the PMSI. However, metastatic stages were excluded from the analyses, and it is the type of lung resection rather than TNM stage that influences 30-day mortality and severe postoperative complications [9].

Certain other relevant variables were not available in our data, including the American Society of Anesthesiologists (ASA) score, ECOG performance status, smoking history and forced expiratory volume in one second (FEV1). However, we accounted for pre-existing pulmonary disease, which usually correlates with impaired FEV1. We also used the modified CCI, which has been validated as a preoperative risk score. The body mass index (BMI) is not included in our database, but we included obesity in the metabolic disease variable.

Our data source does not provide information on the number of beds in the surgical and intensive care unit, nurses, medical practitioners and surgeries performed by residents. Neither does it contain data on the organisation of the surgical team, which could potentially influence the quality of care, such as tumour board meetings, adherence to guidelines and surgeon experience. Indeed, in the PMSI national database, it is not possible to identify the different French practitioners who performed surgeries or to ascertain their experience or their type (e.g., general surgeon vs. thoracic surgeon). However, in France, hospitals are authorised by the authorities to perform this surgery, and most of the surgeons who perform it are qualified in thoracic surgery. Patient quality of life is another essential indicator that cannot be measured using PMSI data.

Further studies would thus be useful in order to take into account additional and more detailed clinical information. In France, this type of study could be conducted using the Epithor database of the French Society of Thoracic and Cardiovascular Surgery [26]. It is also important to be able to take into account multimodal approaches encompassing all phases of care, such as the ERAS (Enhanced Recovery After Surgery) program [27], which opens the way to evaluating enhanced recovery after surgery in the improvement of postoperative complications and mortality in lung cancer surgery. These complementary studies could also focus on benign illnesses, such as pneumothorax, chronic pain, or air leaks, or on length of hospital stay.

These results cannot necessarily be extrapolated directly to other countries. To do so, similar studies would have to be conducted, provided that comparable data are available, in order to take into account differences between healthcare systems and organizations, and in medical ethics.

## 5. Conclusions

This study shows that there are differences in practice between hospitals in each French region. However, interregional variations in the standardised complication rate were moderate. The significant influence of the volume of activity on the occurrence of severe post-operative complications and mortality raises questions about the need to restructure the offer of care for complex surgeries such as LC surgery.

While the authorisation threshold for LC cancer surgery was recently raised to 40 procedures/year, our findings suggest that this threshold should be raised to at least 100 procedures/year if France wants to catch up with other European countries in terms of quality of results.

## Figures and Tables

**Table 1:** Distribution of Clavien–Dindo classification, especially post-operative complications and 30-day mortality (Clavien–Dindo > 2).

	2019	2020	2021	2022	2023
	n = 12,367	n = 11,792	n = 12,542	n = 13,376	n = 14,227
None	6528 (52.8)	6298 (53.4)	6972 (55.6)	7594 (56.8)	7969 (56.0)
Clavien–Dindo ≤ 2	1863 (15.1)	1764 (15.0)	1911 (15.2)	2039 (15.2)	2331 (16.4)
Clavien–Dindo > 2					
Clavien–Dindo IIIa	1308 (10.6)	1207 (10.2)	1178 (9.4)	1264 (9.4)	1375 (9.7)
Clavien–Dindo IIIb	638 (5.2)	652 (5.5)	634 (5.1)	668 (5.0)	748 (5.3)
Clavien–Dindo IVa	337 (2.7)	361 (3.1)	408 (3.3)	440 (3.3)	497 (3.5)
Clavien–Dindo IVb	1412 (11.4)	1240 (10.5)	1196 (9.5)	1151 (8.6)	1088 (7.6)
Clavien–Dindo V	281 (2.3)	270 (2.3)	243 (1.9)	220 (1.6)	219 (1.5)

(Percentage).

**Table 2:** Description of the number of annual procedures for hospitals in each region.

Region	Number of Hospitals	Number of Procedures				
		p10	p25	Median	p75	p90
ARA	24	10	10	50	87	106
BFC	5	10	20	35	43	281
BRE	13	10	35	70	97	128
COR	2	16	16	20	23	23
CVL	8	10	23	44	68	122
GE	13	10	56	121	139	218
HdF	12	10	20	71	108	222
IdF	19	10	11	54	135	327
NA	17	10	27	41	96	138
NOR	8	10	18	42	135	261
OCC	17	10	27	53	87	238
PACA	19	10	17	51	101	229
PdL	14	10	10	43	56	113

Auvergne-Rhones-Alpes (ARA), Bourgogne-Franche-Comté (BFC), Bretagne (BRE), Corse (COR), Centre Val-de-Loire (CVL), Grand-Est (GE), Hauts-de-France (HdF), Ile-de-France (IdF), Nouvelle Aquitaine (NA), Normandie (NOR), Occitanie (OCC), Provence-Alpes-Côte d’Azur (PACA), Pays de la Loire (PdL).

**Table 3:** Number of hospitals in each region according to their level of performance.

Regions	Quantiles of the Standardised Severe Complication (Clavien–Dindo > 2) Rate			
	<23%	23–26%	27–35%	>35%
ARA	7	6	7	4
	29.17	25.00	29.17	16.67
BFC	1	2	2	0
	20.00	40.00	40.00	0.00
BRE	7	2	3	1
	53.85	15.38	23.08	7.69
CVL	1	1	4	2
	12.50	12.50	50.00	25.00
COR	0	0	0	2
	0.00	0.00	0.00	100.00
GE	2	0	5	6
	15.38	0.00	38.46	46.15
HdF	5	1	4	2
	41.67	8.33	33.33	16.67
IdF	5	3	2	9
	26.32	15.79	10.53	47.37
NOR	6	1	0	1
	75.00	12.50	0.00	12.50
NA	5	2	6	4
	29.41	11.76	35.29	23.53
OCC	7	5	1	4
	41.18	29.41	5.88	23.53
PdL	8	2	2	2
	57.14	14.29	14.29	14.29
PACA	3	5	5	6
	15.79	26.32	26.32	31.58

Auvergne-Rhones-Alpes (ARA), Bourgogne-Franche-Comté (BFC), Bretagne (BRE), Corse (COR), Centre Val-de-Loire (CVL), Grand-Est (GE), Hauts-de-France (HdF), Ile-de-France (IdF), Nouvelle Aquitaine (NA), Normandie (NOR), Occitanie (OCC), Provence-Alpes-Côte d’Azur (PACA), Pays de la Loire (PdL).

**Table 4:** Hierarchical logistic regression: Adjusted Odds Ratio of number of annual procedures and type of hospital on the risk of severe complication (Clavien–Dindo > 2).

	aOR	95% CI	*p*-Value
Number of annual procedures			0.0001
<100	1		
101–250	0.83	0.77–0.89	
>250	0.85	0.77–0.93	
Type of hospital			0.0001
Non-academic hospital	1		
Academic (teaching) hospital	0.98	0.89–1.08	
Non-profit private hospital	1.35	1.19–1.52	
Private hospital	1.10	1.01–1.19	
Inter-regional variance	0.034	0.015–0.077	

95% CI: 95% confidence interval.

## Data Availability

The use of the data from the French hospital database by our department was approved by the National Committee for data protection. We are not allowed to transmit these data. PMSI data are available for researchers who meet the criteria for access to these French confidential data (this access is submitted to the approval of the National Committee for data protection) from the national agency for the management of hospitalization (ATIH—Agence technique de l’information sur l’hospitalisation) Address: Agence technique de l’information sur l’hospitalisation 117 boulevard Marius Vivier Merle—69329 Lyon Cedex 03.

## Supplementary Materials

The following materials can be downloaded at https://www.mdpi.com/article/10.3390/cancers17040617/s1, Figure S1. Distribution of the median observed rate and standardised rate of severe complications (Clavien–Dindo > 2) in metropolitan regions; Table S1. Classification of Complications according to Clavien–Dindo; Table S2. Hierarchical logistic regression on the risk of severe complication (Clavien–Dindo > 2); Table S3. Limited resection, Hierarchical logistic regression: Adjusted Odds Ratio of number of annual procedures and type of hospital on the risk of severe complication (Clavien–Dindo > 2); Table S4. Lobectomy, Hierarchical logistic regression: Adjusted Odds Ratio of number of annual procedures and type of hospital on the risk of severe complication (Clavien–Dindo > 2).
