# Supplementary material for: Evaluation of the Quality of Results of Lung Cancer Surgery in France Using the PMSI National Database
Source: Cancers (Basel). 2025 Feb 11;17(4):617. doi: 10.3390/cancers17040617 (PMC11852714; doi:10.3390/cancers17040617)
Supplement: Supplementary file 1 [file cancers-17-00617-s001.zip › cancers-3332742-supplementary.pdf]

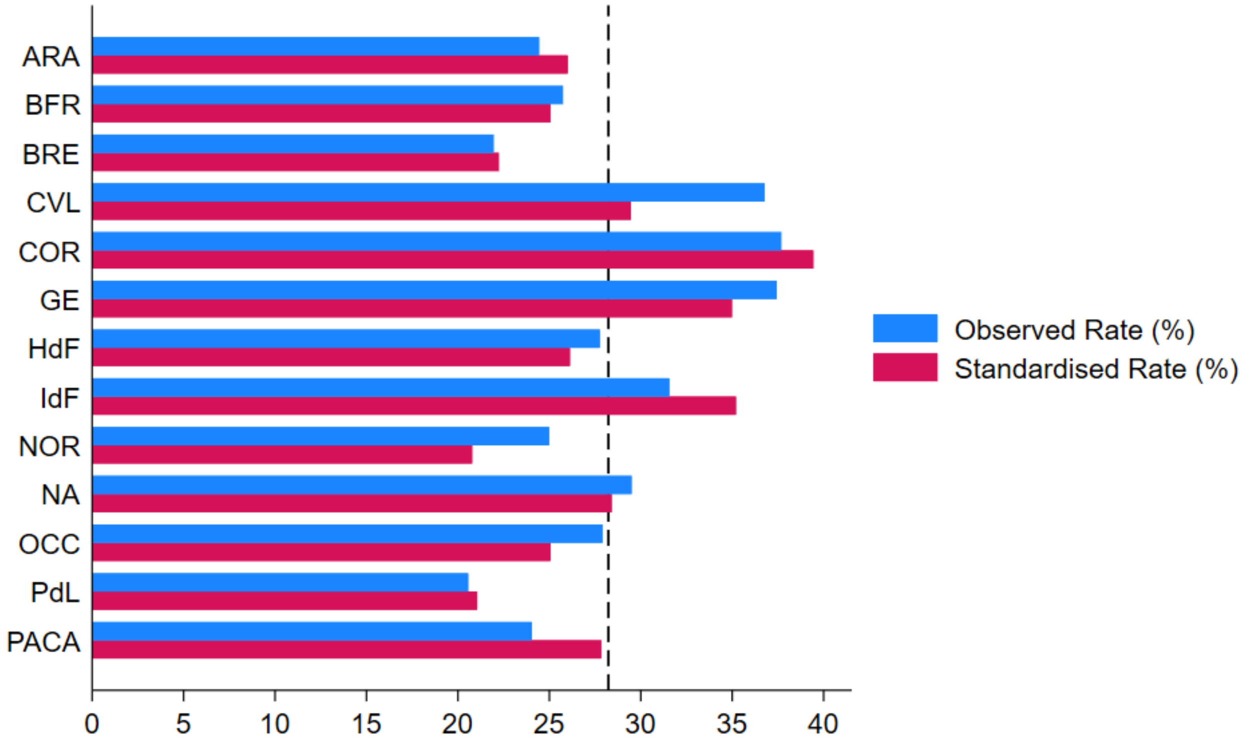


Auvergne-Rhones-Alpes (ARA), Bourgogne-Franche-Comté (BFC), Bretagne (BRE), Corse (COR), Centre Val-de-Loire (CVL), Grand-Est (GE), Hauts-de-France (HdF), Ile-de-France (IdF), Nouvelle Aquitaine (NA), Normandie (NOR), Occitanie (OC), Provence-Alpes-Côte d'Azur (PACA), Pays de la Loire (PdL)

**Figure S1.** Distribution of the median observed rate and standardised rate of severe complications (Clavien-Dindo > 2) in metropolitan regions

**Table S1.** Classification of Complications according to Clavien-Dindo

| Grade | Definition |
|---|---|
| Grade I | Any complication without need for pharmacologic treatment or other intervention |
| Grade II | Any complication that requires pharmacologic treatment or minor intervention only. |
| Grade III | Any complication that requires surgical, radiologic, endoscopic intervention, or multitherapy. |
| Grade IIIa | Intervention does not require general anesthesia. |
| Grade IIIb | Intervention requires general anesthesia. |
| Grade IV | Any complication requiring intensive care unit management and life support |
| Grade IVa | Single organ dysfunction |
| Grade IVb | Multiorgan dysfunction |
| Grade V | Any complication leading to the death of the patient. |

**Table S2.** Hierarchical logistic regression on the risk of severe complication (Clavien-Dindo > 2)

| | Odds ratio | Low CI OR | Upper CI OR | P_value |
|---|---|---|---|---|
| Pulmonary disease | 3.386046 | 3.214915 | 3.566286 | 0.0001 |
| Heart disease | 1.967327 | 1.847898 | 2.094474 | 0.0001 |
| Peripheral vascular | 1.711523 | 1.580256 | 1.853695 | 0.0001 |
| Neurological disease | 3.047553 | 2.727736 | 3.404868 | 0.0001 |
| Liver disease | 0.9659183 | 0.7448855 | 1.252539 | 0.794 |
| Renal disease | 0.95411 | 0.8368032 | 1.087861 | 0.483 |
| Metabolic disease | 0.9305458 | 0.8648582 | 1.001222 | 0.054 |
| Anemia | 2.489786 | 2.333023 | 2.657081 | 0.0001 |
| Infectious disease | 2.717243 | 1.586779 | 4.653081 | 0.0001 |
| Hematological disease | 1.046292 | 0.9364657 | 1.168998 | 0.424 |
| Other disease | 1.137388 | 1.064592 | 1.215161 | 0.0001 |
| Other treatment | 0.8774282 | 0.8074521 | 0.9534687 | 0.002 |
| Extended resection | 1.124866 | 1.028461 | 1.230308 | 0.01 |
| sleeve | 1.193174 | 1.011556 | 1.4074 | 0.036 |
| Pulmonary resection | | | | |
| Lobectomy | 1.024568 | 0.9463376 | 1.109264 | 0.549 |
| Bilobectomy | 1.20888 | 1.034403 | 1.412787 | 0.017 |
| Pneumonectomy | 1.522687 | 1.332401 | 1.740149 | 0.0001 |
| Charlson score | | | | |
| 1 | 0.8478639 | 0.779247 | 0.9225229 | 0.0001 |
| 2 | 0.8417936 | 0.7698518 | 0.9204583 | 0.001 |
| ≥3 | 0.8283458 | 0.7633065 | 0.8989269 | 0.0001 |
| sex | | | | |
| Female | 1.039175 | 0.9880645 | 1.09293 | 0.135 |
| Age (year) | 1.004204 | 1.001426 | 1.00699 | 0.003 |
| Approach | | | | |
| VATS | 0.8170524 | 0.7698586 | 0.8671392 | 0.0001 |
| Robotic | 0.797464 | 0.7264093 | 0.8754691 | 0.0001 |
| Number annual procedure | | | | |
| 101-250 | 0.8316933 | 0.7753189 | 0.8921667 | 0.0001 |
| >250 | 0.8466428 | 0.7664942 | 0.9351721 | 0.001 |
| Type of hospital | | | | |
| Academic | 0.980149 | 0.8908075 | 1.078451 | 0.681 |
| Private non-profit | 1.348554 | 1.196556 | 1.51986 | 0.0001 |
| Private for-profit | 1.101698 | 1.015022 | 1.195775 | 0.021 |
| Intercept | 0.0173533 | 0.0134384 | 0.0224087 | 0.0001 |
| Inter-regional variance | | | | |
| | 0.0336786 | 0.014664 | 0.0773492 | |

**Table S3. Lobectomy,** Hierarchical logistic regression: Adjusted Odds Ratio of number of annual procedures and type of hospital on the risk of severe complication (Clavien-Dindo > 2).

| | aOR | 95%CI | p-value |
|---|---|---|---|
| Number of annual procedures | | | |
| <100 | 1 | | |
| 101-250 | 0.84 | 0.78-0.91 | 0.001 |
| >250 | 0.85 | 0.76-0.96 | |
| Type of hospital | | | |
| Non-academic hospital | 1 | | |
| Academic (teaching) hospital | 1.0 | 0.90-1.12 | 0.0001 |
| Non-profit private hospital | 1.37 | 1.20-1.57 | |
| Private hospital | 1.14 | 1.04- 1.25 | |
| Inter-regional variance | .045 | 0.015-0.14 | |

**Table S4. Limited resection,** Hierarchical logistic regression: Adjusted Odds Ratio of number of annual procedures and type of hospital on the risk of severe complication (Clavien-Dindo > 2).

| | aOR | 95%CI | p-value |
|---|---|---|---|
| Number of annual procedures | | | |
| <100 | 1 | | |
| 101-250 | 0.80 | 0.66-0.97 | 0.06 |
| >250 | 0.78 | 0.60-1.02 | |
| Type of hospital | | | |
| Non-academic hospital | 1 | | |
| Academic (teaching) hospital | 0.79 | 0.61-1.03 | 0.14 |
| Non-profit private hospital | 0.97 | 0.68-1.38 | |
| Private hospital | 0.96 | 0.77-1.20 | |
| Inter-regional variance | .045 | 0.015-0.14 | |
